# Kv1.3 regulation of brain immune function *in vitro* and *in vivo*

**DOI:** 10.1002/alz.093042

**Published:** 2025-01-03

**Authors:** Christine A Bowen, Hai Minh Nguyen, Young Qingyang Lin, Pritha Bagchi, Aditya Natu, Hailian Xiao, Claudia Espinosa‐Garcia, Prateek Kumar, Heike Wulff, Nicholas T Seyfried, Srikant Rangaraju

**Affiliations:** ^1^ Emory University, Atlanta, GA USA; ^2^ University of California Davis, Davis, CA USA; ^3^ Emory University School of Medicine, Atlanta, GA USA; ^4^ Emory University School of Medicine, New Haven, CT USA; ^5^ Yale University School of Medicine, New Haven, CT USA

## Abstract

**Background:**

In neurodegenerative disease such as Alzheimer’s disease and stroke, the brain transitions to pro‐inflammatory profile, where microglia and T‐cells in the brain have increase inflammatory profiles, along with increased Kv1.3 potassium channel abundance. Pharmacological blockade of Kv1.3 *in vivo* reduces Aβ pathology and dampens pro‐inflammatory function. To identify Kv1.3 channels regulation of neurodegenerative models, we examined the protein‐protein interactome of Kv1.3 channels *in vitro*, and Kv1.3 deletion *in vivo*.

**Method:**

TurboID, a biotin ligase, was fused to N‐term or C‐term of Kv1.3 and stably‐transduced in BV2 microglial cells and Jurkat T‐cells. Mass spectrometry (MS) of biotinylated proteins, under resting and lipopolysaccharide (LPS)‐treated conditions, was performed to identify N and C‐terminal interactomes. *Kcna3*(Kv1.3)‐floxed mice were generated and crossed to CMV‐Cre mice to produce a Kv1.3 KO mouse. Kv1.3 KO mice were then exposed to an LPS challenge for 4 days or (middle cerebral artery occlusion) MCAO. Changes in transcriptomic levels were evaluated via RNA‐seq of microglia and brain.

**Result:**

Proximity‐based proteomics of BV2‐Kv1.3‐TurboID microglia identified distinct N‐term (n = 991) and C‐term Kv1.3 (n = 849) interactors. Kv1.3 N‐terminus interacts with plasma membrane proteins (e.g. Cars1, Psma2) and mitochondrial trafficking proteins (TIMM23 and 50). The C‐terminal interactors are modified by LPS‐stimulation (C3, STAT1, OASL1) (n = 36), and dependent on a PDZ‐binding motif (SNX3, ND3, n = 70). Jurkat T‐cell N‐terminal interactors are ER‐processing and plasma membrane (*e.g*. TXNL1, ACTN3, CASP3), whereas C‐terminal interactors are protein processing (*e.g*. EHD1, VAMP2). About 400 proteins are shared between the T‐cell and microglial Kv1.3 interactors. Kv1.3 KO mice have reduced Kv1.3 mRNA and channels in brain and microglia. PCA and differential expression highlight microglial inflammatory LPS response and an MCAO, which is reduced in brain overall. Further analysis comparing Kv1.3 KO and WT will be evaluated.

**Conclusion:**

We identified novel N and C‐terminal domain‐specific and context‐dependent interactors of Kv1.3 channels in immune cells. While the N‐terminus regulates protein processing and localization, the C‐terminus regulates immune signaling during LPS‐stimulation. Some interactions are dependent on the PDZ‐binding domain. KO of Kv1.3 impacts microglial signatures under neuroinflammatory states. microglial transcripts. Identifying microglial Kv1.3 influence in AD may provide knowledge on future biomarkers and treatments.

